# LXR Deficiency Confers Increased Protection against Visceral *Leishmania* Infection in Mice

**DOI:** 10.1371/journal.pntd.0000886

**Published:** 2010-11-16

**Authors:** Kevin W. Bruhn, Chaitra Marathe, Ana Cláudia Maretti-Mira, Hong Nguyen, Jacquelyn Haskell, Thu Anh Tran, Veena Vanchinathan, Upasna Gaur, Mary E. Wilson, Peter Tontonoz, Noah Craft

**Affiliations:** 1 Department of Medicine, Division of Dermatology and Infectious Diseases, Harbor-University of California Los Angeles Medical Center and Los Angeles Biomedical Research Institute, Torrance, California, United States of America; 2 Department of Pathology, Molecular Biology Institute, Howard Hughes Medical Institute, University of California Los Angeles, Los Angeles, California, United States of America; 3 Departments of Internal Medicine, Epidemiology and Microbiology, University of Iowa and the Veterans Affairs Medical Center, Iowa City, Iowa, United States of America; New York University School of Medicine, United States of America

## Abstract

**Background:**

The liver X receptors (LXRs) are a family of nuclear receptor transcription factors that are activated by oxysterols and have defined roles in both lipid metabolism and cholesterol regulation. LXRs also affect antimicrobial responses and have anti-inflammatory effects in macrophages. As mice lacking LXRs are more susceptible to infection by intracellular bacteria *Listeria monocytogenes* and *Mycobacterium tuberculosis*, we hypothesized that LXR might also influence macrophage responses to the intracellular protozoan parasite *Leishmania chagasi/infantum*, a causative agent of visceral leishmaniasis.

**Methods and Findings:**

Surprisingly, both LXRα knock-out and LXRα/LXRβ double-knock-out (DKO) mice were markedly resistant to systemic *L. chagasi/infantum* infection compared to wild-type mice. Parasite loads in the livers and spleens of these animals were significantly lower than in wild-type mice 28 days after challenge. Bone marrow-derived macrophages from LXR-DKO mice infected with *L. chagasi/infantum in vitro* in the presence of IFN-γ were able to kill parasites more efficiently than wild-type macrophages. This enhanced killing by LXR-deficient macrophages correlated with higher levels of nitric oxide produced, as well as increased gene expression of IL-1β. Additionally, LXR ligands abrogated nitric oxide production in wild-type macrophages in response to infection.

**Conclusions:**

These observations suggest that LXR-deficient mice and macrophages mount antimicrobial responses to *Leishmania* infection that are distinct from those mounted by wild-type mice and macrophages. Furthermore, comparison of these findings to other intracellular infection models suggests that LXR signaling pathways modulate host antimicrobial responses in a complex and pathogen-specific manner. The LXR pathway thus represents a potential therapeutic target for modulating immunity against *Leishmania* or other intracellular parasites.

## Author Summary

*Leishmania spp.* are protozoan single-cell parasites that are transmitted to humans by the bite of an infected sand fly, and can cause a wide spectrum of disease, ranging from self-healing skin lesions to potentially fatal systemic infections. Certain species of *Leishmania* that cause visceral (systemic) disease are a source of significant mortality worldwide. Here, we use a mouse model of visceral *Leishmania* infection to investigate the effect of a host gene called LXR. The LXRs have demonstrated important functions in both cholesterol regulation and inflammation. These processes, in turn, are closely related to lipid metabolism and the development of atherosclerosis. LXRs have also previously been shown to be involved in protection against other intracellular pathogens that infect macrophages, including certain bacteria. We demonstrate here that LXR is involved in susceptibility to *Leishmania*, as animals deficient in the LXR gene are much more resistant to infection with the parasite. We also demonstrate that macrophages lacking LXR kill parasites more readily, and make higher levels of nitric oxide (an antimicrobial mediator) and IL-1β (an inflammatory cytokine) in response to *Leishmania* infection. These results could have important implications in designing therapeutics against this deadly pathogen, as well as other intracellular microbial pathogens.

## Introduction

Liver X receptors (LXRs) are a family of nuclear transcription factors that play an integral role in both lipid metabolism and the regulation of inflammation [1], [2]. Two isoforms of LXR exist in both mouse and human: LXRα is mainly expressed in metabolically active tissues such as liver, intestine, kidney and adipose tissue, in addition to macrophages and myeloid dendritic cells (DCs) [3]; LXRβ has a relatively ubiquitous expression pattern [4]. LXRs form functional heterodimers with retinoid X receptors (RXRs) that are activated upon binding to intracellular oxysterols, thus functioning as sensors of cellular cholesterol levels. Upon activation, LXR-RXR complexes promote expression of genes involved in cholesterol efflux, absorption, conversion to bile acids, and lipogenesis [5]. In addition to controlling these key elements of lipid homeostasis, activated LXR also inhibits the development of inflammatory pathways via repression of NF-κB signaling, particularly in macrophages [6]. This dual ability to promote cholesterol efflux and inhibit inflammation supports a protective function for the LXRs against diseases such as atherosclerosis, which are characterized by cholesterol-laden foam cells and chronic inflammation [1]. Consistent with this model, treatment with synthetic LXR agonist molecules reduces disease incidence in animal models of atherosclerosis [7]. LXR-deficient mice, conversely, develop enlarged, cholesterol-laden livers and elevated serum cholesterol upon exposure to high cholesterol diets, and are highly prone to atherosclerosis [8].

In addition to their involvement in the development of these chronic metabolic diseases, macrophages play a fundamental role in innate immune activity. The expression of LXR in macrophages, combined with the ability of these receptors to regulate inflammatory pathways, suggest a role for LXR in combating specific microbial pathogens. Previously it has been demonstrated that LXR-deficient mice are more susceptible than wild-type mice to infection with the intracellular bacterial pathogens *Listeria monocytogenes* [9] and *Mycobacterium tuberculosis* [10]. The increased susceptibility to *Listeria* was associated with an LXR-regulated gene expressed in macrophages called SPα, identified as having anti-apoptotic function. LXR-deficient mice displayed increased macrophage apoptosis during the course of *Listeria* infection, correlating with a decreased ability to clear the bacteria. Demonstration that LXR-associated pathways impact macrophage responses to an intracellular bacterium suggested that these receptors and their downstream metabolic networks might also influence immunity against other microbial pathogens.

The genus *Leishmania*, comprised of numerous distinct species of trypanosomatid protozoa, infects predominantly macrophage cells in their mammalian hosts. *Leishmania* species, particularly *L. major*, have been studied in detail over many decades, helping to define many aspects of host immunity, including initial characterizations of T_H_1- versus T_H_2-type, CD4^+^ T cell responses [11]. Invasion of phagocytic host cells occurs through complex interactions between *Leishmania* surface structures and phagocytic receptors in the host cell [12], which are organized within cholesterol-containing lipid rafts in the outer membrane. Upon cellular entry, promastigotes remain in endosomal structures, transform into amastigotes, multiply, and inhibit immune-mediated clearance by the host through various mechanisms of immunosuppression [13]. Infected macrophages and other immune cells express important proinflammatory cytokines and molecules that play roles in parasite resistance, including IL-1, IL-6, IL-12, TNF-α, CD40L, and IFN-γ [14]–[16]. Also critical to *Leishmania* immunity is the expression of inducible nitric oxide synthase (iNOS) by macrophages, which leads to microbicidal killing via NO production. This pathway is particularly essential in murine cells [17]–[19]. Experimental vaccination regimens in mice that augment immunity against *Leishmania* challenge often enhance these pathways, and correlate with increases in parasite killing.

In this study we examine the role of LXR activity in resistance to the visceralizing species *L. chagasi/infantum*, which replicates primarily in the liver and spleen following intravenous inoculation in the mouse. Given similarities in pathogenesis of infection between *Leishmania* and the bacteria *Listeria* (liver tropism, macrophage invasion, intracellular replication, and requirement for T_H_1 cellular responses for clearance), we hypothesized that LXR might also prove important for *Leishmania* resistance. In contrast to our published *L. monocytogenes* results, we demonstrate here that LXR-deficient mice are more resistant to *L. chagasi/infantum* infection compared to wild-type mice. This suggests that loss of LXR function does not simply lead to a global, general defect in immunity to intracellular pathogens, and broadens the implications of LXR modulation in medical therapies. Here we characterize the increased resistance of LXR-deficient mice to visceral *Leishmania* infection, and present data implicating the role of the macrophage in this phenotype.

## Materials and Methods

### Mice

Mice were housed in a temperature-controlled room under a 12-hour light/12-hour dark cycle and maintained on standard chow under specific-pathogen-free conditions. LXRαβ^+/+^(wild-type), LXRα^−/−^, LXRβ^−/−^, and LXRαβ ^−/−^ (DKO) mice were maintained in a pure C57Bl/6 background, following greater than 20 backcrosses. Knockout and wild-type mice in a mixed background (Sv129xC57Bl/6) were originally provided by David Mangelsdorf (University of Texas Southwestern Medical Center, Dallas, TX) and have been separately backcrossed with each other since their original creation in 1999. Mice were sex- and age-matched with littermates for all *Leishmania* challenge experiments. Mice ranged in age from 6–12 weeks at the beginning of experiments. Typical experiments included equal numbers of both male and female mice, but no significant differences in infection levels were observed between male and female mice in these experiments. All mouse experiments were approved by the Institutional Animal Care and Research Advisory Committee of UCLA, and the Institutional Animal Care and Use Committee at Los Angeles Biomedical Research Institute.

### Leishmania and in vivo infection

*Leishmania chagasi/infantum* (MHOM/BR/00/1669), originally isolated from a patient with visceral leishmaniasis in Northeast Brazil, was maintained by serial intracardiac injection in outbred male golden hamsters. Revirilized amastigotes were isolated from hamster spleens removed from animals infected approximately 3 months prior to euthanasia. Promastigote parasites were grown in hemoflagellate-modified minimal essential medium (HOMEM) [20] at 26°, and passaged for no longer than 6 weeks. To enrich for metacyclic promastigotes, parasites were initially seeded at 3×10^6^ cells/mL and grown for 6 days until cultures reached stationary phase (5−7×10^7^ cells/mL). Parasites grown in this way displayed similar infection kinetics, in both mice and macrophages, to parasites enriched for metacyclics by Ficoll gradient purification, in our hands. Parasites were washed in PBS, counted, and resuspended at 5×10^7^/mL for tail vein injection of 1×10^7^ parasites/200 µL. Following specified durations of infection, mice were euthanized, and parasite burdens were assessed microscopically by blinded counting of Giemsa-stained liver imprints. LDU (Leishman-Donovan units) equals the number of amastigotes per cell nuclei × liver weight (in mg).

### Macrophage derivation and *in vitro* infection

Bone marrow-derived macrophages (BMDM) from C57Bl/6 wild-type or LXR-deficient mice were obtained by differentiating bone marrow cells in complete DMEM/20% FBS supplemented with M-CSF as described [21]. After 7 days, BMDM were collected following trypsinization and 1×10^6^ cells were allowed to adhere overnight to coverslips in 12-well plates for infection assays. Primary peritoneal macrophages were harvested from euthanized animals following thioglycollate injection as described [22]. For CFSE labeling of parasites, *L. chagasi/infantum* promastigotes were incubated in 5 µM CFSE in PBS for 10 min. at 37°, and washed twice in PBS. Cells were infected with *L. chagasi/infantum* promastigotes (grown to stationary phase to enrich for metacyclics, as above) at multiplicity of infections (MOI) ranging from 1∶1 to 7∶1. Infections were performed in serum-free media, and plates centrifuged briefly to synchronize infection. After 1 hour of infection, extracellular parasites were removed by rinsing twice with PBS, and cells were incubated in fresh medium, at 37°, 5% CO_2_ for an additional hour. Macrophage infection levels were quantitated by microscopy by Giemsa staining cover slips and counting parasites. A minimum of either 500 amastigotes or mononuclear cell nuclei were counted per slide, and numbers reported as amastigotes/nuclei. Alternatively, infected macrophages were removed from plates, fixed in 2% paraformaldehyde/PBS, and analyzed by flow cytometry for CFSE (FL-1) positive events.

### Nitric oxide assays

Primary macrophages were derived in culture as above. Nitric oxide production was measured following infection of 1×10^6^ macrophages in 12-well plates with *L. chagasi/infantum* at an MOI of 5∶1 in complete DMEM medium. Designated wells were preincubated with 10 U/ml recombinant murine IFN-γ overnight for 16–24 hours prior to infection. At varying time points after initiating infection, supernatants were sampled and assayed for the accumulation of nitrite ion, which is proportionate to nitric oxide production, using a Griess Reagent System (Promega). Samples were analyzed by absorbance photometry at a wavelength of 535 nm and nitrite ion concentration calculated by comparison with a standard curve of serial dilutions of sodium nitrite.

### Leishmania load determination by quantitative PCR

Total genomic DNA was extracted from frozen liver and spleen samples (<25 mg) from infected animals, using an UltraClean Tissue DNA Isolation Kit (Mo Bio Laboratories, Inc., Carlsbad, CA) according to the manufacturer's instructions. Quantitative PCR assays were performed using TaqMan probes and TaqMan Universal PCR Master Mix (Applied Biosystems, Inc.), with typical 25 µL reactions containing 200 nM of each primer and probe and 2 µL of genomic DNA template diluted 1∶10 following column elution. *L. chagasi/infantum* parasite DNA was detected using primers and probe specific for GP63, a known virulence gene [23]. Quantitative values of *Leishmania* were normalized to levels of a mouse gene (TNF-α) to account for differences in amounts of tissue sampled. All primers and probes were synthesized by Integrated DNA Technologies (Coralville, Iowa); sequences available upon request. PCRs were performed on an Applied Biosystems Prism 7000 sequence detector, and data was analyzed using SDS1.2.3 software. GP63 Ct values were converted to absolute parasite counts using previously determined standard curves, and were normalized to the amount of tissue DNA in each sample as determined by TNF-α Ct values.

### Real-time RT-PCR to assess gene expression

Total RNA was extracted from tissues or cells growing *in vitro* using TRIzol reagent (Invitrogen) and was reverse transcribed to obtain cDNA (Applied Biosystems). SYBR-based or Taqman-based real-time quantitative PCR assays were performed using an Applied Biosystems 7900HT sequence detector as previously described [24]. Data was normalized to housekeeping genes RPLP0 (36B4) or HPRT1, both of which were externally verified as appropriate reference genes under these experimental variables. Primer sequences are available upon request.

### Statistics

Differences between mean values of counted Giemsa-stained parasites, parasite loads measured by qPCR, and NO levels were analyzed by a two-tailed Student's t-test.

## Results

### LXR-deficient mice have decreased parasite burden following *L. chagasi/infantum* infection

**Figure 1:**
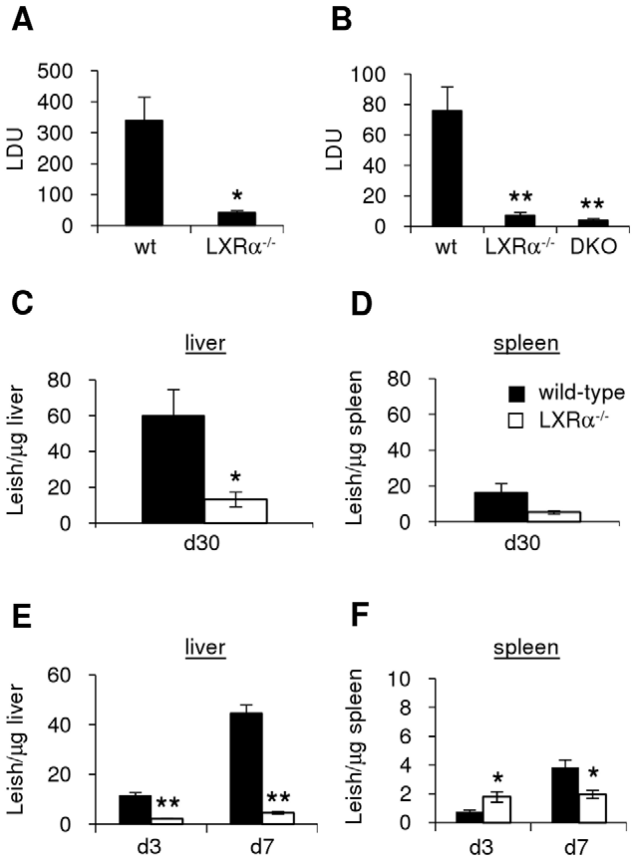
LXR-deficient mice display enhanced resistance to Leishmania infection of the liver and spleen. Wild-type or LXR-deficient C57Bl/6 mice (except for (B), which were the mixed Sv129xC57Bl/6 background) were challenged with 1×10^7^ live, stationary-phase *L. chagasi/infantum* parasites, and livers and spleens harvested at various time points. A–B. Parasite loads in the livers at d28 were quantitated by microscopy. Leishman-Donovan Units (LDU)  =  [(# amastigotes/# mononuclear cells) multiplied by liver weight in mg]. C–F. Parasite loads in liver (C, E) and spleen (D, F) were quantitated by qPCR of genomic DNA from wild-type (black bars) and LXRα^−/−^ (white bars) tissue. Levels are expressed as # parasites/µg tissue. Groups consisted of 7–9 mice/group for A–D, 5 mice/timepoint for E–F. Results are representative of at least 3 independent experiments. Error bars represent standard error; * denotes p<0.05, ** p<0.005, p-value for Fig. 1D  = 0.06, comparisons to wild-type.

We have previously reported that LXRα^−/−^ and LXRαβ^−/−^ (LXR-DKO) mice are more susceptible to *Listeria monocytogenes* infection than wild-type control mice [9]. To determine the role of LXR in the pathogenesis of the macrophage-infecting *Leishmania* parasites, we challenged these same knockout mouse strains with the visceralizing species *Leishmania chagasi/infantum*. Because of the high expression levels of LXRα in the liver, we chose to study this visceral infection model of *L. chagasi/infantum*, as it is found predominantly in the liver and spleen of mice following intravenous injection. Wild-type C57Bl/6 mice display maximal numbers of *L. chagasi/infantum* in the liver four weeks after intravenous challenge infection with stationary-phase promastigotes, followed by subsequent parasite clearance associated with granuloma formation [25]. Parasite numbers in the spleen, in contrast, gradually increase over time and can persist chronically over the life of the animal [26]. We infected wild-type and LXRα^−/−^ C57Bl/6 mice intravenously with virulent, stationary-phase *L. chagasi/infantum* promastigotes, and assessed parasite burden in the liver by microscopy four weeks later, when levels peak in wild-type mice. Mice deficient for LXRα had significantly decreased parasite burdens compared to wild-type animals 28 days following challenge (Fig. 1A, p = 0.007). LXRβ^−/−^ mice, compared to wild-type mice, did not exhibit reproducible differences in parasite load in two experiments (data not shown), and were not examined further for this study. To determine if these results were mouse strain-dependent, we also challenged wild-type and LXR-deficient mice in a mixed strain background, Sv129xC57Bl/6. All mice, both wild-type and LXR-knockout, had been backcrossed within this mixed strain for many years and were therefore congenic. Although mixed background wild-type mice generally had lower parasite loads than C57Bl/6 wild-type mice, LXRα^−/−^ and LXR-DKO mice in the mixed background also displayed significantly lower parasite loads than their wild-type littermates (Fig. 1B, LXRα^−/−^: p = 0.003, LXR-DKO: p = 0.003).

To assess the lower parasite levels in the spleen and at earlier time points after infection, we utilized quantitative PCR (qPCR). Liver and spleen genomic DNA was harvested and subjected to Taqman-based qPCR using *Leishmania*-specific primers and probes. In agreement with microscopy counts, parasite numbers were reduced in livers of LXRα^−/−^ animals compared to wild-type mice one month after infection (Fig. 1C, p = 0.014). In addition, parasite numbers measured by qPCR were also reduced in LXRα^−/−^ spleens (Fig. 1D, p = 0.06). To determine the level of *Leishmania* infection in LXR-KO mice at earlier time points, we measured parasite levels in these tissues at 3 and 7 days following intravenous challenge with *L. chagasi/infantum*. The amount of parasite DNA detected in LXRα^−/−^ livers was significantly lower on both day 3 and day 7 than the amount detected in wild-type livers (Fig. 1E, d3: p = 0.002, d7: p = 0.0002). Spleens displayed relatively low overall levels of parasite DNA at early time points, but by day 7, parasite loads in wild-type spleens were significantly higher than in LXRα^−/−^ spleens (Fig. 1F, d3: p = 0.04, d7: p = 0.02). Taken together, the differences in organ parasite loads in LXR-deficient mice suggest that LXR-regulated pathways can significantly influence the overall control of visceral leishmaniasis infections.

### LXR-deficient macrophages clear parasites more efficiently than wild-type macrophages in the presence of IFN-γ *in vitro*

**Figure 2:**
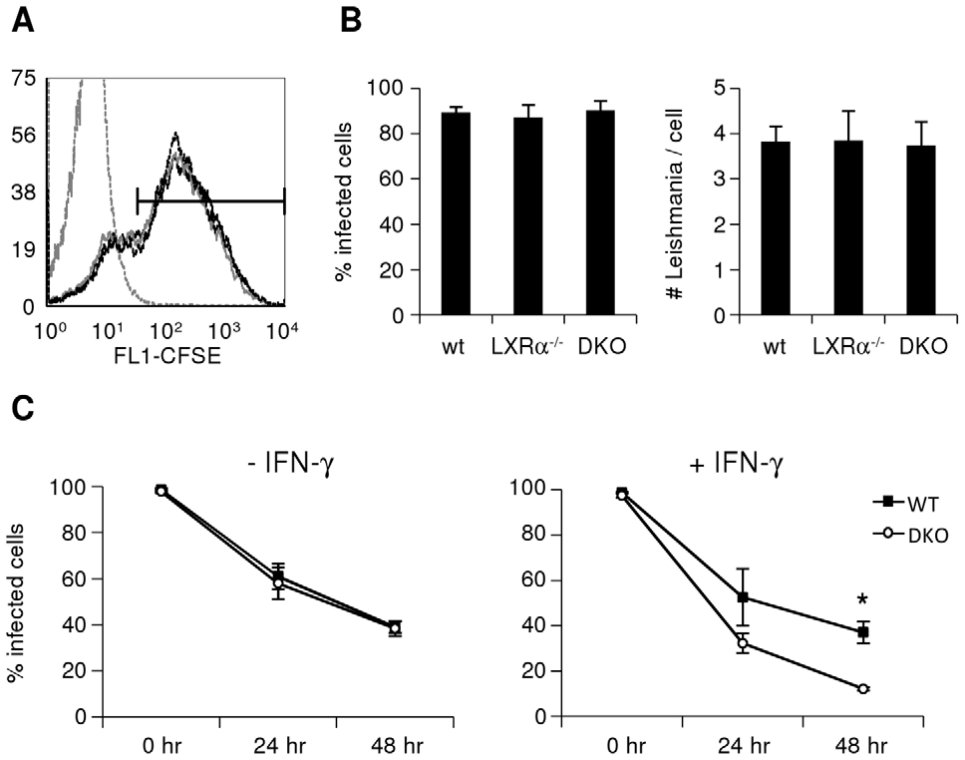
LXR-deficient macrophages are infected similarly, but kill Leishmania more efficiently than wild-type macrophages treated with IFN-γ. Bone marrow-derived macrophages (BMDM) were infected with stationary-phase, (A) CFSE-labeled or (B) unlabelled *L. chagasi/infantum* at an MOI of 10∶1 for 1 hour, and 2 hours later percentages of infected cells (CFSE-positive events) were determined by (A) flow cytometry (WT uninfected  =  gray dotted line; WT infected  =  black solid line; LXRα^−/−^ infected  =  gray solid line; LXR-DKO infected  =  black dotted line) or (B) microscopy (the number of parasites per cell was also assessed by microscopy). C. BMDM were infected with stationary-phase, unlabelled *L. chagasi/infantum* at an MOI of 20∶1 for 2 hours in the presence or absence of IFN-γ (overnight pretreatment), and immediately, 24, or 48 hours later were Giemsa-stained for microscopic quantitation of numbers of amastigotes per cell. Similar results were found in least 3 independent experiments; error bars represent standard error; * denotes p<0.01.

Given the high expression levels of LXRα in macrophages, and the central role these cells play in *Leishmania* pathogenesis, we examined the effect of macrophage LXR deficiency on the kinetics of parasite uptake and infection *in vitro*. We differentiated bone marrow-derived macrophages (BMDM) *in vitro* for 7 days, then infected these macrophages with stationary-phase *L. chagasi/infantum* promastigotes for 1 hour, before removing any extracellular parasites by washing. Parasites were labeled with the intracellular dye CFSE to allow for analysis of infected macrophages by flow cytometry. Two hours following infection, macrophages from wild-type, LXRα^−/−^, and DKO mice all displayed similar patterns of CFSE staining (Fig. 2A), suggesting that parasite uptake was similar in all cells. We also infected wild-type and LXR-deficient macrophages grown on cover slips with *L. chagasi/infantum*. Giemsa staining and microscopy also revealed identical percentages of infected cells, as well as identical numbers of parasites/cell in wild-type, LXRα^−/−^, and DKO macrophages (Fig. 2B). These results suggest that LXR does not affect the process of initial parasite internalization by macrophages *in vitro*.

We next measured the kinetics of infection and parasite clearance over time. When macrophages were infected with *Leishmania* alone, there was no difference between wild-type and DKO macrophages in the percentage of infected cells over 48 hours (Fig. 2C, left panel). During *in vivo* infections, however, macrophage killing of parasites is known to be dependent on endogenous IFN-γ produced by other cell types, including NK cells and T cells [16]. We thus repeated *in vitro* infections by pretreating macrophages with exogenous IFN-γ, in order to provide a relevant macrophage activating stimulus. In the presence of IFN-γ, DKO macrophages displayed more efficient clearance of internalized parasites over 48 hours than wild-type macrophages (Fig. 2C, right panel; p = 0.007 at 48 hr). Interestingly, IFN-γ did not appear to enhance parasite clearance in wild-type macrophages. These results suggest that LXR-dependent pathways in macrophages partially inhibit the IFN-γ-mediated clearance of *Leishmania*.

### Macrophages from LXR-DKO mice have enhanced NO secretion

**Figure 3:**
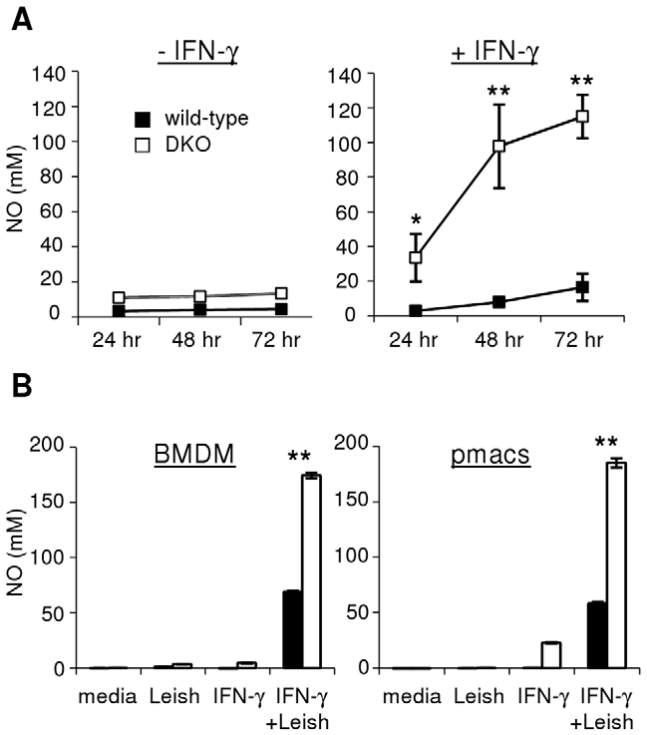
LXR-deficient macrophages produce increased levels of NO following exposure to Leishmania and IFN-γ in vitro. A. BMDM from individual wild-type and DKO (Sv129xC57Bl/6) mice were stimulated *in vitro* with stationary-phase *L. chagasi/infantum* promastigotes for the times indicated, in the absence (left) or presence (right) of IFN-γ pre-treament. B. BMDM (left) or peritoneal macrophages (right) from wild-type (solid bars) or LXR-DKO (empty bars) mice (C57Bl/6 background) were infected with stationary-phase *L. chagasi/infantum* (with or without IFN-γ pre-treatment) for 24 hours *in vitro*. Supernatants were removed and levels of NO were quantitated by the Griess method. Error bars represent standard deviations of triplicate NO measurements. Results are representative of at least 3 independent experiments; * denotes p<0.05, ** p<0.005.

We next examined the effect that LXR deficiency has on specific parasite defense mechanisms of macrophages. Expression of inducible nitric oxide synthase (iNOS) by macrophages leads to nitric oxide (NO) production resulting in destruction of intracellular pathogens [27]. We measured NO production by macrophages following *Leishmania* infection in the presence or absence of IFN-γ *in vitro*. Without exogenous IFN-γ, levels of NO secreted by BMDM infected with *Leishmania* parasites over three days remained low (Fig. 3A, left panel). However, when BMDM were pretreated with IFN-γ before parasite infection, cells from LXR-DKO mice secreted enhanced levels of NO compared to wild-type cells (Fig. 3A, right panel). This finding was verified in both BMDM and peritoneal macrophages (Fig. 3B). The increased level of NO production by DKO macrophages in the presence of IFN-γ correlates with enhanced clearance of the parasite (Fig. 2C), suggesting that increased IFN-γ-induced NO production by macrophages may contribute to the enhanced resistance of LXR-deficient mice to *Leishmania* infection.

### LXR ligands inhibit *Leishmania*-induced NO from wild-type macrophages

**Figure 4:**
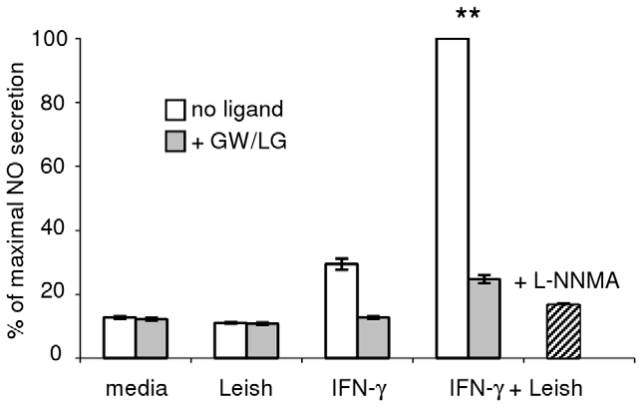
NO production by macrophages stimulated with IFN-γ + Leishmania is inhibited by LXR ligands. BMDM were preincubated overnight with LXR (GW  =  GW3965) and RXR (LG  =  LG268) ligands, with or without IFN-γ, prior to a 1 hour *in vitro* infection with stationary-phase *L. chagasi/infantum*. Amount of NO secreted in 24 hours by wild-type BMDM stimulated with *IFN-γ + Leishmania* in the absence of ligand was normalized to 100%. L-NNMA is a NOS inhibitor. Error bars represent standard deviations of triplicate NO measurements. Results are representative of at least 3 independent experiments; ** denotes p<0.005.

We hypothesized that if activated LXR complexes are functioning to downregulate NO production following induction with IFN-γ/*Leishmania*, then the addition of LXR-activating ligands to wild-type cells might result in decreased levels of NO *in vitro*. Since wild-type BMDM secrete measurable amounts of NO in response to IFN-γ/*Leishmania*, we tested the ability of LXR agonists to inhibit this production. We pre-exposed wild-type BMDM to the synthetic LXR/RXR ligands GW3965 (GW) and LG268 (LG) with or without IFN-γ overnight prior to incubation of cells with *Leishmania* for one hour. The NO produced in wild-type cells in response to IFN-γ/*Leishmania* was significantly reduced (by 75%) in cells pretreated with LXR/RXR ligands (Fig. 4). Pretreatment with L-NNMA, a broad NOS inhibitor, had similar effects on NO production as GW/LG pretreatment.

### Gene expression following *Leishmania* infection in LXR-deficient mouse liver and spleen, and macrophages

**Figure 5:**
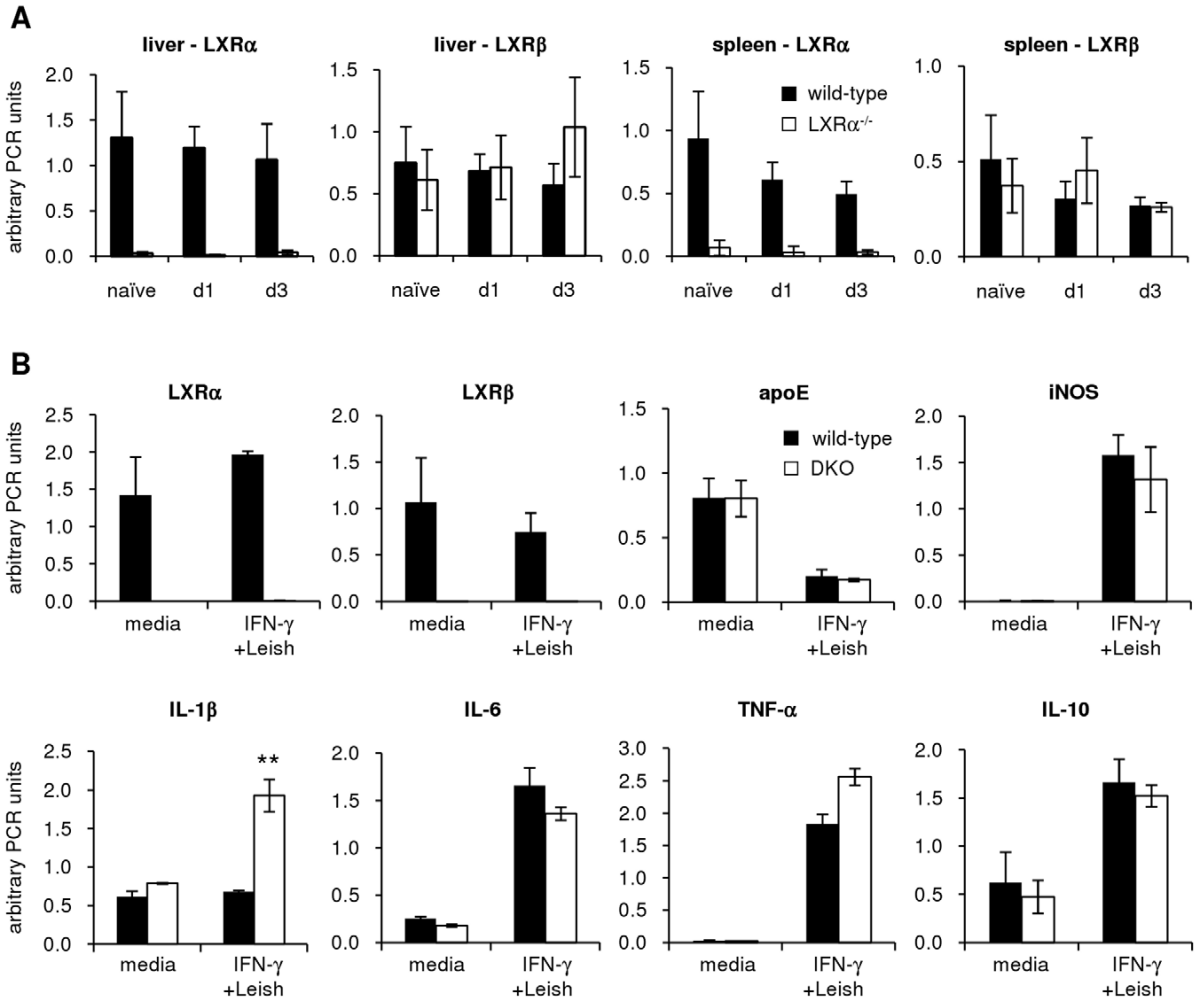
Gene expression in wild-type and LXR-deficient tissues and macrophages following Leishmania infection. A. Wild-type and LXRα-deficient mice were intravenously challenged with 1×10^7^ live, stationary-phase *L. chagasi/infantum* parasites, and RNA was prepared from livers and spleens 24 and 72 hours following infection. Real-time RT-PCR was performed with SYBR primers specific for LXRα and LXRβ. B. BMDM were preincubated overnight with IFN-γ, infected with stationary-phase *Leishmania* for 1 hour, washed to remove extracellular parasites, and incubated for an additional 2 hours. RNA was harvested, and levels of various genes were determined by real-time RT-PCR. Error bars represent standard deviations of replicate infections; ** denotes p<0.005. Results are representative of at least 3 independent experiments.

We assessed the effect of *Leishmania* infection on mouse tissue gene expression *in vivo*, and in macrophages *in vitro*. Wild-type and LXRα-KO mice were infected with *L. chagasi/infantum* 24 or 72 hours before sacrificing, and RNA was harvested from livers and spleens. LXRα expression in either tissue did not change upon infection of wild-type mice (as expected, LXRα was not expressed in LXRα-KO mice), and LXRβ was not induced by *Leishmania* infection in either wild-type or LXRα-KO mice (Fig. 5A). In accordance with previously published microarray data in human and mouse macrophages [14], [28], expression of both LXRα and LXRβ was unaltered by *Leishmania* infection of IFN-γ-pretreated BMDM (Fig. 5B). These results suggest that the *Leishmania* resistance observed in LXR-deficient mice cannot be attributed to a lack of *Leishmania*-induced upregulation of the LXR genes. We examined a prototypical LXR-regulated gene, apoE, which contains an LXR responsive element in its promoter, and is known to be upregulated upon LXR activation [29]. Baseline expression levels of apoE, as well as expression following *Leishmania* infection (which was decreased), were similar in wild-type and LXR-DKO macrophages (Fig. 5B). We also examined macrophage expression levels of several genes known to be involved in resistance to visceral *Leishmania*, including iNOS, IL-1β, IL-6, TNF-α, and IL-10. Wild-type and DKO BMDM expressed similar levels of iNOS RNA in response to IFN-γ and *Leishmania* exposure (Fig. 5B), suggesting that the observed differences in NO production by wild-type compared to LXR-deficient macrophages (Fig. 3) are not due to changes in iNOS transcription. Of the cytokine genes examined, IL-1β was significantly induced by IFN-γ and *Leishmania* infection in LXR-deficient macrophages, but not in wild-type macrophages. No other genes examined were differentially regulated in the absence of LXR, including IL-12 and IFN-γ, which were detected only at very low levels by RT-PCR (data not shown). These results suggest that IL-1β is one possible cytokine that might contribute to LXR control of *Leishmania* resistance during infection.

## Discussion

The LXR nuclear receptors regulate both lipid homeostasis and inflammation in mammalian cells. LXR has emerged as a potential drug target for chronic metabolic and inflammatory diseases such as atherosclerosis. Macrophages are central in these processes, and the expression and activation of both LXR isoforms in macrophages have been well established. Since macrophages are innate immune effectors, we and others have begun to investigate the relationship between these metabolic regulatory genes and the ability to mount responses against microbial pathogens. We demonstrate here an unexpected enhanced resistance of LXR-deficient mice to infection with the intracellular protozoan *Leishmania chagasi/infantum*, a parasite that resides and replicates predominantly in macrophage phagolysosomes. The *in vivo* resistance in mice lacking LXRα was evident in the liver as early as three days following inoculation, and continued over one month. In accordance with this resistance phenotype, we report that LXR-deficient macrophages pre-exposed to IFN-γ and infected with *L. chagasi/infantum in vitro* had fewer intracellular parasites after 48 hours and produced increased levels of NO compared to their wild-type counterparts. In addition, IFN-γ/*Leishmania*-induced NO production by wild-type macrophages could be inhibited by exposure to LXR ligands. Whereas the *in vivo* resistance phenotype was apparent in both LXRα^−/−^ and LXR-DKO (LXRαβ ^−/−^) mice, the *in vitro* phenotypes were only consistently observed in LXR-DKO macrophages, which may reflect different compensatory abilities of LXRβ *in vitro* versus *in vivo*. Overall, we hypothesize that the significantly decreased parasite burden in LXR-deficient mice might be broadly attributed to 1) changes in host cell susceptibility to parasite invasion, establishment, and/or persistence, and/or 2) differences in functional host immune responses following parasite establishment within macrophages.

The enhanced resistance of both LXRα^−/−^ and DKO mice to *L. chagasi/infantum* was surprising in light of several recent findings documenting these genotypes' decreased immunity to various infections. Our group previously reported that LXR-deficient mice are acutely susceptible to infection with the bacterium *Listeria monocytogenes* [9]. *Listeria*, like *Leishmania*, resides intracellularly in mammalian host cells (including macrophages), and requires T_H_1-mediated immune responses for clearance. The absence of LXRα leads to increased apoptosis of macrophages in response to *Listeria* infection, a phenotype attributable in part to decreased expression of an LXR-regulated, anti-apoptotic factor called SPα [9]. We did not observe increased apoptosis levels in LXR-deficient BMDM compared to wild-type macrophages following *Leishmania* infection *in vitro*. One major difference between the two organisms is that *Listeria*, unlike *Leishmania*, escape the phagolysosomal compartment and enter the host cell cytoplasm. Both *Listeria* and *Shigella flexneri*, a gram-negative intracellular bacterium that also has a cytoplasmic niche, strongly induce LXRα expression in murine BMDM, in contrast to extracellular bacteria [9]. Addition of the NOD2 ligand muramyl dipeptide (MDP) to BMDM also induces expression of LXRα mRNA, suggesting that specific intracellular signaling pathways initiated by NOD-like receptors (NLRs) in the cytoplasm trigger upregulation of LXRα. These pathways are likely not directly activated by *Leishmania* in macrophages, as the parasite normally remains sequestered in phagosomal compartments. Our results (Figure 5), and microarray data of gene expression patterns in both human and mouse macrophages, suggest that *Leishmania* infection does not affect LXR expression levels or LXR-mediated gene expression [14], [28].

LXR deficiency in mice also results in increased susceptibility to the intracellular pathogen *Mycobacterium tuberculosi*s [10]. Mice lacking both isoforms of LXR had decreased clearance of mycobacterial load from the lungs, spleen and liver over several weeks of infection. This defect correlated with a reduction of lung-infiltrating neutrophils in mice lacking LXRα, along with reduced neutrophil-attracting chemokines. Although typical pro-inflammatory/microbicidal mediators TNF-α and iNOS remained at basal levels following infection in both wt and KO mice, IL-12p40 levels were reduced in LXRα KO mice. Interestingly, a significant increase of arginase mRNA was observed in DKO mice, although it was unclear if this was specific for either isoform, ArgI or ArgII.

The metabolic state of macrophages can directly influence intracellular growth and/or survival of intracellular amastigotes. Arginine metabolism is regulated by LXR and has previously been implicated in *Leishmania* pathogenesis. The ArgII gene is induced greater than 50-fold upon ligand activation of LXRα, and its arginase enzyme product functions to hydrolyze the amino acid arginine to urea and ornithine [30]. Promotion of this reaction might influence *Leishmania* survival within host macrophages in two distinct ways. First, the products of the arginase pathway are precursors for polyamine synthesis that positively affect parasite growth and metabolism [31], [32]. Removal of LXRα might lead to a reduction in these small metabolites that are normally utilized by amastigotes, leading to inhibited growth and increased susceptibility to oxidative stress. Secondly, nitric oxide synthases (NOS) compete with arginase enzymes for the utilization of L-arginine as a substrate, alternatively converting it into NO and L-citrulline. Previous experiments in our group have demonstrated that overexpression of ArgII in RAW macrophages *in vitro* leads to an inhibition of nitrite production in response to inflammatory stimuli including LPS, poly I:C, PGN, LTA and CpG [30]. Decreased ArgII expression in LXR-deficient cells, conversely, might be predicted to lead to an increase in NO production, due to higher levels of arginine metabolite. We were unable to detect significant levels of ArgII in wild-type macrophages upon *Leishmania* infection, or in mice infected with *Leishmania* (data not shown), consistent with the absence of overt upregulation of LXR by infection. However, the exact role of arginase activity, and its impact on parasite survival in LXR-deficient animals, will require further investigation to define.

Specific genes known to be suppressed by activated LXR include pro-inflammatory cytokines induced by LPS and bacterial stimuli, including iNOS, cyclooxygenase (COX)-2, and IL-6 [6]. Early innate cytokine responses by macrophages and neutrophils determine in part whether *Leishmania* infections resolve, with Type I immune responses generally correlating with control of infections [25]. We demonstrate here that mRNA levels of IL-1β were higher in LXR-DKO macrophages than in wild-type macrophages following IFN-γ pretreatment and *L. chagasi* infection *in vitro*. However, we did not observe differences in macrophage expression levels of several other cytokines potentially involved in *Leishmania* resistance (including IL-6, TNF-α, and IL-10), nor in levels of iNOS. Enhanced expression of the pro-inflammatory cytokine IL-1β by macrophages might partially explain the early resistance of LXR-deficient mice, which displayed lower liver parasite load as early as three days following challenge. It is clear, however, that other cell types in addition to macrophages contribute to the global leishmanial resistance phenotype *in vivo*. Bensinger et. al. recently demonstrated a link between LXR signaling and lymphocyte proliferation, whereby loss of LXRβ led to enhanced T cell responses to antigenic challenge [33]. The effects of LXR deficiency on other cells, including T_H_1 CD4^+^ T cells, in terms of *Leishmania* immunity and cytokine expression, are currently being studied in our model. Additionally, LXR signaling was also recently implicated in the clearance of apoptotic cells by macrophages, and the maintenance of immune tolerance [34]. The relevance of this finding to the observed pathogen immune phenotypes remains to be defined.

The LXR-regulated network of genes affects many aspects of metabolism and inflammation, and has been extensively studied with hopes of positively influencing metabolic disorders such as hyperlipidemia and atherosclerosis. Mechanisms by which LXR pathways impact immune function will be important to consider when targeting these molecules for activation in cardiovascular disease. With regards to leishmaniasis, understanding how pathogenesis is impacted by LXR signaling may lead to novel interventions in this disease process as well. Since LXRs down-modulate inflammatory pathways, blocking LXR activation or expression might augment key cell-mediated immune responses necessary for *Leishmania* killing. The observation, however, that LXR-deficient mice, while more resistant to visceral *Leishmania* infection, are more susceptible to bacterial infections caused by *Listeria monocytogenes* and *Mycobacterium tuberculosis*, suggests a complex immune phenotype that is pathogen-dependent. Targeting LXR or LXR-mediated genes for antagonism may nonetheless represent a feasible strategy to modulate immunity against certain types of intracellular pathogens like *Leishmania*. Continued study into the role of LXR in resistance to specific microorganisms may also lead to enhanced understanding of complex host-pathogen relationships.
